# Social Withdrawal (Hikikomori) Conditions in China: A Cross-Sectional Online Survey

**DOI:** 10.3389/fpsyg.2022.826945

**Published:** 2022-03-14

**Authors:** Xinyue Hu, Danhua Fan, Yang Shao

**Affiliations:** Shanghai Mental Health Center, Shanghai Jiao Tong University School of Medicine, Shanghai, China

**Keywords:** social withdrawal, hikikomori, online survey, diagnosis, China

## Abstract

**Objective:**

A form of pathological social withdrawal which is also called hikikomori has been proved its existence in China. But the prevalence and characteristics of hikikomori in China remain unknown. Past studies had investigated the hikikomori phenomenon in three cities of China. The purpose of this study is to discover the prevalence of hikikomori in a convenient online sample in China as well as the difference in demographic characteristics and other possible traits between hikikomori sufferers and the general population.

**Methods:**

A total of 1,066 youths (mean age = 22.85 years) in China completed the online questionnaire, which consisted of questions about demographics, the 25-item Hikikomori Questionnaire (HQ-25), the Internet Addiction Test (IAT), the Loneliness Scale (UCLA), and the General Health Questionnaire (GHQ). SPSS is used to evaluate the data.

**Results:**

Of the 1,066 youths, 980 (91.9%) were identified as belonging to group A (be not social isolation nor withdrawn), 46 (4.3%) to group B (marked social isolation in one's home or withdrawn with a duration of at least 3 months), and 40 (3.8%) to group C (marked both social isolation in one's home and withdrawn with a duration of at least 3 months). The hikikomori group (combined group B and group C) accounted for 8.1%. The present data suggest that residence and loneliness are related to the occurrence of hikikomori. HQ-25 score of the hikikomori group was significantly higher than the comparison group. The UCLA score showed that those in the hikikomori group felt lonelier than those in the comparison. The regression model predicted hikikomori risk (χ^2^ = 38.658, *P* = 0.000), the Hosmer-Lemeshow test value is 7.114 and *P* = 0.524 > 0.05.

**Conclusion:**

The grouping criterion in our present study is reasonable and such a grouping criterion can screen out potential populations of hikikomori. When people develop into hikikomori sufferers in the present, their social withdrawal behaviors and feeling of loneliness are both much more severe than in the past. The possible relationships between hikikomori and loneliness reflect the need to give the youths more social support, to help them connect with society.

## Introduction

Social withdrawal syndrome was first described in Japan as hikikomori and defined as the state of confining oneself to one's home for more than 6 months and strictly limiting communication with others (Teo and Gaw, 2010). Kato, Kanba, and Teo proposed diagnostic criteria for hikikomori that addressed this to some degree by classifying hikikomori who infrequently leave their home on a spectrum of severity and classifying those who do not yet meet the 6-month criterion as “pre-hikikomori” (Tan et al., 2021). Initially, hikikomori was seen as a phenomenon unique to Japanese society, but more recently similar cases have been reported in many other countries, including Western ones (Malagón-Amor et al., 2018). Cases were reported in the United States (Teo et al., 2015), Oman (Sakamoto et al., 2005), South Korea (Lee et al., 2013), Hong Kong (Chan and Lo, 2016), India (Teo et al., 2015), France (Guedj-Bourdiau, 2011), and Italy (Ranieri, 2015). Besides these case reports, some surveys showed that hikikomori exists in other countries besides Japan, especially in urban areas, such as Australia, Bangladesh, Iran, Thailand, and Taiwan (Kato et al., 2019). According to past research methods, the method of questionnaire survey is widely used in the past studies of hikikomori.

In behavioral terms, hikikomori comprises 3–6 months or more of self-imposed real-life home isolation, characterized by the avoidance of social and family relationships (while online relationships may exist), and occupational and educational disengagement (Teo et al., 2015; Tajan et al., 2017), and has been associated with loneliness, social deficits, and other psychopathology (Uchida and Norasakkunkit, 2015; Stip et al., 2016). There are reports of hikikomori coexisting with some psychiatric disorders (Kondo et al., 2013). For instance, similar hikikomori-like behaviors are observed during the depression phase of bipolar disorder (Kato et al., 2019). Hikikomori was also related to elevated levels of current loneliness and depressive symptoms (Bowker et al., 2019). Hikikomori onset is typically signified by adolescent school refusal, progressing to full real-life withdrawal in young adulthood (Teo and Gaw, 2010; Devylder et al., 2020). Those with hikikomori (compared to those without) are more likely to utilize the virtual environment to escape, which may exacerbate real-life deficits (Uchida and Norasakkunkit, 2015; Stip et al., 2016). The virtual world is appealing, increasingly accessible, and can be used excessively by those with hikikomori over their offline reality (Wong et al., 2015; Stip et al., 2016). So some scholars proposed comorbidity with Internet addiction (Kato et al., 2012; De Michele et al., 2013; Lee et al., 2013).

Hikikomori seems to be a transcultural condition (De Michele et al., 2013; Stip et al., 2016), which has proved the existence of hikikomori. A prospective cohort study was conducted in Hongkong to follow-up on 104 participants on their changes in social, psychological, and physical health as exposed to the hikikomori lifestyle (Yuen et al., 2019). Participants were interviewed at baseline, 6 and 12 months by administering a set of questionnaires and anthropometric measurements. All three health domains of hikikomori were significantly improved over the follow-up period as evidenced by increased social network scores, decreased perceived stress scores, and reduced blood pressure levels and waist-to-hip ratios. Almost half of the participants have recovered from hikikomori by returning to the workforce in society; however, the health improvements were dominant in those that remained as hikikomori and were associated with the gradual swapping of exercise practices from light to moderate level strength (Yuen et al., 2019).

One past study has proved the existence of hikikomori among young people in three urban areas of China (Shanghai, Beijing, and Shenzhen), among these participants who completed the survey, 13 (9.5%) were identified as belonging to the withdrawal group, 7 (5.1%) to the asocial group, and 9 (6.6%) to the hikikomori group (both withdrawn and asocial for more than 3 months) (Liu et al., 2018). Another research found that when compared to comparison participants, hikikomori was more likely to be male, have less offline social capital, have visited a mental health clinic, and have suicidal ideation. Physically isolated only individuals were more likely to be at higher risk of experiencing risk behaviors, including unwanted pregnancy and debt, and were more likely to be socially disengaged and experience less care from their father. Hikikomori in China seems to exhibit a higher risk of suicidal ideation and psychopathology, while physically isolated individuals suffer from developmental difficulties, experience more risky behaviors, and have poor family relationships (Wong et al., 2017).

However, the criteria of hikikomori are not uniform. Criteria proposed by Kato, Kanba and Teo took 6 months as the time node (Kato et al., 2019). Past studies in three cities took 3 months as the time node (Liu et al., 2018). They divided people with both social withdrawal and social avoidance in one's home for more than 3 months as the hikikomori group. Hence, our study hopes to understand the current situation of hikikomori in China through a larger sample size survey. We expend the sample all around China, trying to explore whether social withdrawal and social avoidance in one's home must exist at the same time and whether there is a difference between the duration of symptoms for 3 and 6 months.

## Materials and Methods

### Participants and Procedure

A total of 1,066 valid online questionnaires were collected. The sample comprised 390 males (36.59%) and 676 females (63.41%), *M*_age_ = 22.85 years, SD = 3.83. Participants were identified as belonging to group A (be not isolation nor withdrawn), to group B (marked social isolation in one's home or withdrawn with a duration of at least 3 months), and to group C (marked both social isolation in one's home and with a duration of at least 3 months).

Participants took part in an online survey launched on WeChat. The specific approach was: to contact the counselors of universities and colleges to send the questionnaire weblink into the WeChat group which teachers and students all in. The range of the questionnaire included all around China, thanks to the help of teachers in colleges and universities. Participants who were interested could access the study online and take part in the study. Also, students can share the weblink with friends they think may be interested in the hikikomori study, or people who may be hikikomori sufferers. Those who agreed to participate answered a range of questions about their social life and their experiences with social withdrawal. All questionnaires that were answered completely and sufficiently were included in the study.

The informed consent form explicitly indicated that participation was voluntary, that respondents were free to withdraw from the study at any time, that if at any point respondents chose to discontinue participation, they were not required to provide any explanation, and there was no penalty associated with withdrawing. Respondents were only able to complete and submit the questionnaire once consent to participate in the study was provided, and they indicated that they understood the nature of the research being conducted.

Online assessments are reliable and valid methods and they can facilitate the study. A correlational study of socioeconomic factors and the prevalence of hikikomori in Japan from 2010 to 2019 had conducted online assessments and found the incidence of hikikomori (Nonaka and Sakai, 2021a). Data collected by recruiting participants from a web-based sample (Nonaka and Sakai, 2021b) and the occurrence of hikikomori in samples of Italian young adults were demonstrated through an online survey (Amendola et al., 2021).

### Materials

#### Hikikomori Symptoms

The 25-item Hikikomori Questionnaire (HQ-25) was developed as a self-administered instrument for assessing the severity of symptoms of hikikomori (Teo et al., 2018). The HQ-25 consisted of 25 questions regarding psychological features and behavioral patterns of the typical hikikomori syndrome, such as lack of social connectedness, active social isolation or withdrawal behavior, avoidance of social contact, and a sense of alienation from society. All items of HQ-25 were rated on a scale ranging from 0 (strongly disagree) to 4 (strongly agree). The HQ-25 has a score range of 0–100. Developers of HQ-25 proposed a cutoff score of 42 (out of 100), which was associated with a sensitivity of 94% and specificity of 61% in their clinical study. In our previous work, we have developed a Chinese version of this scale. The reliability and validity of HQ-25 in China were also tested. The Cronbach's alpha of the scale was 0.946; the Cronbach's alpha of the subscale were 0.917, 0.870, and 0.800. The test-retest reliability of the total scale was 0.932 (Hu et al., 2021). The result of confirmatory factor analysis of the model fitting of HQ-25-Chinese version χ^2^/DF = 7.769, NFI = 0.860, GFI = 0.838, CFI = 0.876, and RMSEA = 0.080^2828^. The sensitivity and specificity of the Chinese version of the HQ-25 in identifying hikikomori (both social isolation in one's home and with a duration of at least 3 months) are 93.48 and 90.74% (true positive rate = 93.48%, true negative rate = 90.74%). This study used a modified version of the definition of hikikomori proposed before (Liu et al., 2018). In brief, the survey inquired about (1) lack of social connectedness and interaction, (2) physical withdrawal, and (3) duration of social withdrawal.

#### Internet Addiction

Internet addiction was measured by Young's Internet Addiction Test (IAT) which has 20 items regarding internet overuse. All items begin with the phrase “How often do you…”, e.g., “How often do you try to cut down the amount of time you spend online and fail?” Respondents are requested to choose one of the following scores: 5 = always, 4 = often, 3 = frequently, 2 = occasionally, and 1 = rarely. The IAT has been used to measure the severity of internet addiction. The total score of IAT ranges from 20 to 100. The Cronbach's alpha of the Chinese version scale was 0.930, the reliability of the Chinese version scale was 0.940 (Lu et al., 2019). In the present study, we classified the level of internet addiction according to the cutoff points previously reported (Young, 1998).

#### Loneliness

Loneliness was measured by asking participants to answer questions from the UCLA Loneliness Scale (Russell, 1996): Do you feel a lack of companionship? Do you feel left out? Do you feel isolated from others? and Do you feel in tune with people around you? The validated four-item version of this scale was used due to space restrictions, to allow participants to rate each item on frequency, intensity, and duration. Participants indicated how often that happened to them on a sliding scale from 1 (never) to 5 (always). The Cronbach's alpha of the Chinese version scale was 0.901, the reliability of the Chinese version scale was 0.910 (Guo et al., 2017).

#### Mental Status

Mental status was measured by the General Health Questionnaire-12 (GHQ-12). GHQ-12 is a popularly used screening self-report for emotional disorders among adults. It includes 6 positive items and 6 negative items. The total score of GHQ-12 ranges from 0 to 36, higher total scores reflect higher levels of psychological morbidity or distress, a lower score means better mental status. Participants whose scores are lower than 13 are at good mental status. The Cronbach's alpha of the Chinese version scale was 0.830, the reliability of the Chinese version scale was 0.608 (Liu et al., 2019).

### Ethics

The studies involving human participants were reviewed and approved by The Research Ethical Committee at Shanghai Mental Health Center. Written informed consent to participate in this study was provided by the participants.

Participants received information about the purpose and design of the study. They were informed that their participation was voluntary and that any participant could withdraw without penalty at any time. They were also informed that their responses would remain anonymous and that only the researchers would see their questionnaires. Only if participants submit the informed consent can participants proceed to the next page to fill out the questionnaires through the designed weblink.

### Statistical Analysis

The data were analyzed using the software package SPSS 26.0 (SPSS Inc., Chicago, IL). The frequencies and means of the demographic variables of the study were first calculated by descriptive statistics. Data were presented as means ± standard deviations.

The differences between demographic variables and hikikomori prevalence among the three groups were analyzed by ANOVAs and chi-squared tests. The results of multiple comparisons were presented when there was a significant statistical difference within the three groups. Bonferroni was used to conduct the *post-hoc* test.

## Results

### Socio-Demographic Characteristics

In total, 1,066 participants completed the survey, among whom 980 (91.9%) were identified as belonging to group A (be not isolation nor withdrawn), 46 (4.3%) to the group B (marked social isolation in one's home or withdrawn with a duration of at least 3 months), and 40 (3.8%) to group C (marked both social isolation in one's home and with a duration of at least 3 months).

First of all, the result showed significant differences between group A, group B, and group C in the score of HQ-25 (*F* = 5.231, *P* = 0.005). This indicates that such a grouping criterion is reasonable and such a grouping criterion can screen out potential populations of hikikomori. Those three groups were significantly different in gender (χ^2^ = 7.850, *P* = 0.020), residence (χ^2^ = 20.804, *P* = 0.002), and educational level (χ^2^ = 13.845, *P* = 0.008) (see Table 1).

**Table 1:** Socio-demographic characteristics of different groups.

	**Total (%) *N* = 1,066**	**A (%)** ***n* = 980**	**B (%)** ***n* = 46**	**C (%)** ***n* = 40**	**χ^**2**^**	***P***	**A vs. B**	**A vs. C**	**B vs. C**
Gender					7.850	0.020^*^	0.017^*^	0.176	0.006^**^
Male	390 (36.6)	362 (36.9)	9 (19.6)	19 (47.5)			<0.05	>0.05	<0.05
Female	676 (63.4)	618 (63.1)	37 (80.4)	21 (52.5)			<0.05	>0.05	<0.05
Registration					4.382	0.112	0.104	0.168	0.907
Urban	696 (65.3)	631 (64.4)	35 (76.1)	30 (75.0)			–	–	–
Rural	370 (34.7)	349 (35.6)	11 (23.9)	10 (25.0)			–	–	–
Residence					20.804	0.002^**^	0.011^*^	0.006^**^	0.922
Home	287 (26.9)	246 (25.1)	21 (45.7)	20 (50.0)			<0.05	<0.05	>0.05
Dormitory	631 (59.2)	595 (60.7)	20 (43.5)	16 (40.0)			<0.05	<0.05	>0.05
Rent	146 (13.7)	137 (14.0)	5 (10.9)	4 (10.0)			>0.05	>0.05	>0.05
Others	2 (0.2)	2 (0.2)	0 (0.0)	0 (0.0)			>0.05	>0.05	>0.05
Only child					0.868	0.648	0.505	0.533	0.353
Yes	586 (55.0)	539 (55.0)	23 (50.0)	24 (60.0)			–	–	–
No	480 (45.0)	441 (45.0)	23 (50.0)	16 (40.0)			–	–	–
Family structure					6.573	0.284	0.187	0.679	0.176
Core	927 (87.0)	855 (87.2)	39 (84.8)	34 (85.0)			–	–	–
Single parent	82 (7.70)	73 (7.4)	6 (13.0)	3 (7.5)			–	–	–
Rebuild	46 (4.30)	44 (4.5)	0 (0.0)	3 (7.5)			–	–	–
Others	11 (1.10)	8 (0.8)	1 (2.2)	0 (0.0)			–	–	–
Education level					13.845	0.008^**^	0.006^**^	0.125	0.451
Below undergraduates	161 (15.1)	139 (14.2)	14 (30.4)	8 (20.0)			<0.05	>0.05	>0.05
Undergraduates	603 (34.6)	553 (56.4)	24 (52.2)	26 (65.0)			>0.05	>0.05	>0.05
Master and above	302 (56.6)	288 (29.4)	8 (17.4)	6 (15.0)			>0.05	<0.05	>0.05
Monthly income					9.530	0.146	–	–	–
<4,000	186 (17.4)	177 (18.1)	7 (15.2)	2 (5.0)			–	–	–
4,000–8,000	310 (29.1)	290 (29.6)	18 (26.1)	2 (20.0)			–	–	–
8,000–12,000	272 (25.5)	246 (25.1)	13 (28.3)	13 (32.5)			–	–	–
>12,000	298 (28.0)	267 (27.2)	14 (30.4)	17 (42.5)			–	–	–
Monthly expenses					10.163	0.118	–	–	–
<1,000	169 (15.9)	156 (15.9)	10 (21.7)	3 (7.5)			–	–	–
1,000–3,000	704 (66.0)	650 (66.3)	27 (58.7)	27 (67.5)			–	–	–
3,000–5,000	138 (12.9)	125 (12.8)	4 (8.7)	9 (22.5)			–	–	–
>5,000	55 (5.2)	49 (5.0)	5 (10.9)	1 (2.5)			–	–	–

* *symbol indicates the value p < 0.05*

** *symbol indicates the value p < 0.001*.

Using *t*-test, participants from single-parent families had significantly higher HQ-25 scores than those core families (*t* = 2.873, *P* = 0.004). Participants with a diagnosis of mental disorders had significantly higher HQ-25 scores than those with no mental disorder diagnosis (*t* = 7.828, *P* = 0.000). There was a significant difference in the education level in the HQ-25 scale score (*F* = 17.188, *P* = 0.000), the score of the below undergraduate group is higher than undergraduate (*t* = 5.603, *P* = 0.000) and master's degrees and above group (*t* = 4.816, *P* = 0.000). There was no significant difference in HQ-25 scores between males and females (*t* = −0.427, *P* = 0.669), between registration of urban and rural (*t* = 1.634, *P* = 0.103), between the only child and the not-only child (*t* = 0.446, *P* = 0.656).

Using the chi-square test, the results showed that the three groups had significant correlations with gender, residence, educational level, and diagnosis of mental disorders. There was no significant correlation with registration, economic sources, monthly income, and whether they were the only child.

### Psychopathology

There were significant differences between group A and group B in HQ-25 score (*t* = 2.737, *P* = 0.006), UCLA score (*t* = 2.452, *P* = 0.014), IAT score (*t* = 2.001, *P* = 0.046). There were significant differences between group A and group C in the UCLA score (*t* = 2.333, *P* = 0.020).

There were significant differences in HQ-25 scores among the three groups (*F* = 5.231, *P* = 0.005), the score of group B is significantly higher than group A (*t* = 2.737, *P* = 0.006), the score of group C is higher but not significantly than group A (*t* = 1.874, *P* = 0.061). There were no significant differences between group B and group C's HQ-25 (*t* = 0.445, *P* = 0.658), UCLA score (*t* = 0.013, *P* = 0.989), IAT score (*t* = 0.422, *P* = 0.674), GHQ score (*t* = 0.640, *P* = 0.524) (see Table 2).

**Table 2:** Scores of HQ-25, UCLA, IAT, GHQ among group A, B, and C.

	**Total** ***N* = 1,066**	**A** ***n* = 980**	**B** ***n* = 46**	**C** ***n* = 40**	***F*** **/χ^2^**	***P***	**A vs. B**	**A vs. C**	**B vs. C**
HQ-25	33.14 ± 18.285	32.61 ± 17.912	40.11 ± 22.796	38.05 ± 19.683	5.231	0.005^**^	0.006^**^	0.065	0.601
UCLA	41.80 ± 10.216	41.50 ± 9.962	45.24 ± 12.939	45.28 ± 11.706	5.393	0.005^**^	0.014^*^	0.020^*^	0.987
IAT	47.02 ± 14.311	46.73 ± 14.094	51.02 ± 16.889	49.53 ± 15.815	2.625	0.073	0.046^*^	0.225	0.628
GHQ	20.67 ± 3.350	20.70 ± 3.213	19.96 ± 4.541	20.60 ± 4.776	1.100	0.333	0.140	0.849	0.374
Mental disorders					4.048	0.046^*^	0.324	0.075	0.728
Yes	1,003, 11.1%	54, 5.5%	4, 8.7%	5, 12.5%			>0.05	>0.05	>0.05
No	63, 5.9%	926, 94.5%	42, 91.3%	35, 87.5%			>0.05	>0.05	>0.05

* *symbol indicates the value p <0.05*

** *symbol indicates the value p <0.001*.

Group B and group C were combined into the hikikomori group (*N* = 86, 8.1%): marked with a duration of at least 3 months, or both social isolation in one's home and withdrawn. Using group A as the comparison group (*N* = 980, 91.9%). The prevalence in our present research is 8.1%. The score of hikikomori group is significantly higher than comparison group in HQ-25 (*t* = 3.193, *P* = 0.001), UCLA score (*t* = 3.286, *P* = 0.001), IAT score (*t* = 2.240, *P* = 0.025).

According to the answer of three questions, we compare these three groups in which answer is “never,” “past,” and “now”, there are significant differences in the score of HQ-25 (*F* = 94.736, *P* = 0.000), UCLA (*F* = 64.074, *P* = 0.000), IAT (*F* = 40.908, *P* = 0.000), and GHQ (*F* = 3.155, *P* = 0.044) among the group “never,” “past,” and “now”. Group “now” are significantly higher than group “never” in the score of HQ-25 (*F* = 32.514, *P* = 0.000), UCLA (*F* = 22.045, *P* = 0.000), IAT (*F* = 6.609, *P* = 0.011), there is no significant difference in the score of GHQ (*F* = 1.101, *P* = 0.296) between “now” and “never” group. Group “now” are significantly higher than group “past” in the score of HQ-25 (*F* = 18.242, *P* = 0.000), UCLA (*F* = 26.744, *P* = 0.000), there are no significant differences in the score of IAT (*F* = 2.342, *P* = 0.127), GHQ (*F* = 1.277, *P* = 0.260) between “past” and “now” group.

According to the duration of social withdrawal, we compare two groups' people whose duration of social withdrawal is 3–6 months and above 6 months, there is no significant difference in the score of HQ-25 (*F* = 0.550, *P* = 0.460), UCLA (*F* = 1.060, *P* = 0.306), IAT (*F* = 1.010, *P* = 0.318) and GHQ (*F* = 0.148, *P* = 0.702) between these two groups.

Using the chi-square test, the results showed that there are no significant differences between the two groups (duration of social withdrawal is 3–6 months vs. duration of social withdrawal is above 6 months) in gender, registration, residence, family structure, whether they were the only child, educational level, and monthly income and expenses, see Table 3.

**Table 3:** Comparison between “3” and “6 months”.

	**3–6 months *n* = 34**	**Above 6 months** ***n* = 52**	***F*** **/χ^2^**	***P***
Gender			0.837	0.360
Male	8 (23.5)	17 (32.7)		
Female	26 (76.5)	35 (67.3)		
Registration			1.057	0.304
Urban	22 (64.7)	39 (75.0)		
Rural	12 (35.3)	13 (25.0)		
Residence			0.566	0.754
Home	16 (47.1)	22 (42.3)		
Dormitory	13 (38.2)	24 (46.2)		
Rent	5 (14.7)	6 (11.5)		
Only child			0.952	0.329
Yes	14 (41.2)	27 (51.9)		
No	20 (58.8)	25 (48.1)		
Family structure			2.100	0.350
Core	30 (88.2)	41 (78.8)		
Single parent	3 (8.82)	5 (9.62)		
Rebuild	1 (2.94)	6 (11.5)		
Others	0 (0.00)	0 (0.00)		
Education level			1.315	0.518
Below undergraduates	12 (35.3)	16 (30.8)		
Undergraduates	15 (44.1)	29 (55.8)		
Master and above	7 (20.6)	7 (13.5)		
Monthly income			2.295	0.513
<4,000	7 (20.6)	9 (17.3)		
4,000–8,000	8 (23.5)	20 (38.5)		
8,000–12,000	12 (35.3)	13 (25.0)		
>12,000	7 (20.6)	10 (19.2)		
Monthly expenses			0.740	0.864
<1,000	8 (23.5)	10 (19.2)		
1,000–3,000	20 (58.8)	29 (55.8)		
3,000–5,000	3 (8.82)	7 (13.5)		
>5,000	3 (8.82)	6 (11.5)		
Mental disorders			0.146	0.780
Yes	7 (20.59)	9 (17.31)		
No	27 (79.41)	43 (82.69)		
HQ-25 score	51.03 ± 18.348	59.98.50 ± 16.741	0.550	0.460
UCLA score	50.24 ± 9.909	54.92 ± 11.322	1.060	0.306
IAT score	56.82 ± 13.588	61.33 ± 15.600	1.010	0.318
GHQ score	21.65 ± 3.123	22.40 ± 2.959	0.148	0.702

### The Influence Factor of Hikikomori

Using binary logistic regression to analyze and explore the factors affecting the hikikomori risk of youth, and introduced gender, educational level, occupation, diagnosis of mental disorders, Internet addiction, mental health condition, loneliness in the model. The results showed that the regression model predicted by arguments of “gender,” “residence,” “only child,” “family structure,” “educational level,” “diagnosis of mental disorders,” “age,” “loneliness,” “internet addiction,” and “mental health condition” predicted hikikomori risk (χ^2^ = 38.658, *P* = 0.000). The Hosmer-Lemeshow test value is 7.114 and *P* = 0.524 > 0.05, indicating that the regression model is well adapted. The Cox-Snell correlation strength value is 0.036 and the Nagelkerke value is 0.083. Among them, the Wald values of “gender,” “residence,” “only child,” “family structure,” “educational level,” “diagnosis of mental disorders,” “age,” “loneliness,” “internet addiction,” and “mental health condition” were 0.947 (*P* = 0.331), 1.763 (*P* = 0.184), 0.502 (*P* = 0.479), 0.715 (*P* = 0.870), 2.234 (*P* = 0.327), 0.469 (*P* = 0.494), 0.009 (*P* = 0.924), 4.807 (*P* = 0.028), 0.623 (*P* = 0.430), and 3.697 (*P* = 0.055), respectively (see Table 4).

**Table 4:** Logistic regression analysis of influencing factors of hikikomori.

	**B**	**SE**	**Wald**	***DF***	***P***	**OR**
Residence			11.539	3	0.038	
Loneliness	0.028	0.013	4.807	1	0.028	1.028

*−2 log-likelihood = 559.188, Cox-Snell R^2^ = 0.036, Nagelkerke R^2^ = 0.083*.

## Discussion

The prevalence of hikikomori has not been reported in Chinese cultures so our present study is significant. Our study is the first one to investigate hikikomori conditions all around China, whose sample size is unprecedented. Another strength of our study is its use of a standardized instrument to screen for hikikomori: Chinese version of 25-item Hikikomori Questionnaire. In total, 1,066 participants completed the survey, among whom 980 (91.9%) were identified as belonging to group A (be not isolation nor withdrawn), 46 (4.3%) to the group B (marked social isolation in one's home or withdrawn with a duration of at least 3 months), and 40 (3.8%) to group C (marked both social isolation in one's home and with a duration of at least 3 months). The hikikomori group (combined group B and group C) accounted for 8.1%. The prevalence in our present research is kind of consistent with past research in three urban areas of China (Shanghai, Beijing, and Shenzhen), in which 13 (9.5%) were identified as belonging to the withdrawal group, 7 (5.1%) to the asocial group, and 9 (6.6%) to the hikikomori group (both withdrawn and asocial for more than 3 months) (Liu et al., 2018). But it seems kind of higher than that in Hong Kong which had rates of 2.5% (more than 6 months) and 2.6% (<6 months) self-perceived social withdrawal among young people aged 12–29 years in Hong Kong (Wong et al., 2015). It may be because our grouping criterion uses 3 months instead of 6 months. Studies in the past have made such recommendations: it is worth considering whether the traditional 6-month criterion for severe social withdrawal may be too long and the duration for early detection and intervention may have been prolonged (Wong et al., 2015).

According to the duration of social withdrawal in our present study, there is no significant difference in the score of HQ-25 (*F* = 0.550, *P* = 0.460) between 3 to 6 months and above 6 months. There are no significant differences in gender, registration, residence, family structure, whether they were the only child, educational level, and monthly income and expenses. Consistent with the previous studies (Wong et al., 2015; Liu et al., 2018; Kato et al., 2019; Tan et al., 2021), this indicates that using 3 months instead of 6 months is reasonable and good for early detection and intervention.

Utilizing this reasonable grouping criterion, we investigated the prevalence of hikikomori in a convenience online sample in China. In our present research, the score of HQ-25 showed significant differences between group A (HQ-25 score = 32.61 ± 17.912), group B (HQ-25 score = 40.11 ± 22.796), and group C (HQ-25 score = 38.05 ± 19.683). Specifically, group A is significantly lower than group B and group C in the HQ-25 score. The score of the hikikomori group (HQ-25 score =39.15 ± 21.306) is significantly higher than the comparison (HQ-25 score =32.61 ± 17.912) group in HQ-25 (*t* = 3.193, *P* = 0.001). This again indicated that such a grouping criterion is reasonable and such a grouping criterion can screen out potential populations of hikikomori. The score of HQ-25 can also help in identifying social withdrawal sufferers, we suggest that a score of 40 can be used as a critical value.

There may be limitations in the participants' population because the specific approach in our study was contacting the counselors of universities and colleges to send the questionnaire weblink into the WeChat group which teachers and students all in. The way of our recruitment methods was to invite counselors to help recruit potential participants, this approach may select more social withdrawal participants and therefore may increase the prevalence of the hikikomori condition, the sample may be not representative enough and (self-)selection bias took place. In fact, both the level of social status, the economic income, and the state of work may be related to social withdrawal. We suggest investigating people of all walks of life in the future. In addition to social status, economic income, and the state of work, age is also a factor worth expanding. The Cabinet Office announced that the estimated number of hikikomori aged between 40 and 65 years is 610,000 in Japan, including individuals over the age of 40 years would increase the number of hikikomori sufferers even further (Japan Cabinet Office, 2019). Hence, we can investigate people of all ages in all walks of life in the future, to explore the relationship between social withdrawal and age, not only in the youth of universities and colleges.

Furthermore, other factors can also be taken into account for further research. A correlational study of socioeconomic factors and the prevalence of hikikomori in Japan from 2010 to 2019 suggest that socioeconomic factors may relate to the increase in hikikomori and that these factors should be considered when identifying the individual or cultural factors that cause hikikomori (Nonaka and Sakai, 2021a). The hikikomori state was related to lower economic status because patients with current hikikomori state significantly frequently received social security than patients with past hikikomori experience and others, which indicates that the present Hikikomori state severely impairs the quality of life of patients (Imai et al., 2021).

Because of the online questionnaire, we asked whether the participants had mental disorders before instead of asking the participants to care about the specific mental illness, so personality disorders, psychotic disorders, and neurodevelopmental disorders were not considered in the screening process. A lack of detailed information regarding mental also limited the description of mental status. The extent to which hikikomori may overlap with existing psychiatric conditions is still unclear. Concerning the diagnosis of mental disorders, since it was an online questionnaire, only whether there had been a diagnosis without more detail. Several studies have attempted to examine this, but they have been inconsistent in screening for the full range of psychiatric disorders. For example, a prevalence study of hikikomori in Japan by Koyama et al. reported that 45.5% of hikikomori did not have a lifetime prevalence of another psychiatric disorder (Koyama et al., 2010). Besides, as a nature of cross-sectional study design, it could not show direct causality.

Our results do show a mixed picture of psychiatric and social factors for severe social withdrawal. The results on one hand indicate that there are increased negative behaviors and higher GHQ-12 scores for social withdrawal individuals, which are proxy correlates of psychiatric concerns. On the other hand, only 5.9% had mental disorders history in the past. These findings are inconsistent with the work carried out in Japan indicating that around 54.5% had experienced a lifetime psychiatric disorder (Koyama et al., 2010). The mental status of anxiety was significantly severer in patients with current and past hikikomori states in the study of psychiatry clinic. It showed that patients with current or past Hikikomori experience felt more anxiety than other patients without hikikomori experience, and the anxiety level of patients with current hikikomori patients tends to be high compared to those with past hikikomori state (Imai et al., 2021). In our present study, group “now” are significantly higher than group “past” in the score of HQ-25 (*F* = 18.242, *P* = 0.000), UCLA (*F* = 26.744, *P* = 0.000), which indicate that social withdrawal in the past can develop into hikikomori sufferers to some degree. When people develop into hikikomori sufferers in the present, their social withdrawal behaviors and feeling of loneliness are both much more severe than in the past.

Other clinical features of psychopathology are associated with hikikomori. For instance, psychotic experiences and delusional experiences are associated with hikikomori (Yasuma et al., 2021). Consistent with research in Italy, clinical features of psychopathology such as aggressive behaviors and substance misuse were more prevalent among hikikomori participants. Moreover, they also found that symptoms of hikikomori showed strong associations with overall personality dysfunction, which highlighted the need to disentangle the intricate relation between hikikomori and psychopathology, and they were discussed considering scientific advances (Amendola et al., 2021). Another study proved the use of instrumental support, behavioral disengagement stress coping skills, self-compassion, and psychological stress were associated with hikikomori behaviors (Nonaka and Sakai, 2021b). We acknowledge that the inclusion of a psychiatric assessment in the study would significantly affect this particular finding. However, we regard this as a very large discrepancy that cannot be explained solely by the presence of a psychiatric assessment factor. Mental disorders can be probably affected by hikikomori because people who are isolated in one particular room are more likely to suffer from other mental diseases.

Nevertheless, such a causal relationship is not clear, we cannot be sure whether the hikikomori results in other mental disorders or not. There is another possibility. People who suffer from some kind of mental disorder may prefer to lock themselves in the room, whether active or passive. For instance, in depression, other than depressed mood, decreased motivation and activity are major symptoms that may present in the form of withdrawal-like outcomes. Similar hikikomori-like behaviors are observed during the depression phase of bipolar disorder. Schizophrenia and psychotic disorders, social anxiety disorder and other anxiety-related disorders, personality disorders, and autism spectrum disorder are also included in these probable mental disorders. This leads us to suspect that social withdrawal among young people in China could be a result of the complex interaction between developmental, social, psychological, and employment challenges in China. Qualitative studies using in-depth interviews are needed to examine what the pull-and-push factors are leading to social withdrawal in China.

The possible relationships between hikikomori and loneliness reflect the need to give the youths more social support, to help them connect with society. To engage and attract those who appear to be unreachable, we can provide online counseling services that offer a secure and distant channel for young people to reestablish their social connection with others. Since socially withdrawn young people are willing to express their distress *via* online platforms such as forums and microblogs, a more proactive approach can be adopted such as commenting on their posts and discussing their problems with them on these online platforms. Professional training of social workers and community service is necessary. We suggest helping hikikomori sufferers have better-coping flexibility and have more chance of being able to self-direct themselves out of a socially withdrawn period.

Hikikomori stands out owing to the extreme levels of isolation and associated impairment. Existing research suggests that people with health problems are more likely to suffer from hikikomori, which means many hikikomori sufferers have additional psychiatric conditions while the extent to which hikikomori may overlap with existing psychiatric conditions is still unclear. Hikikomori can also be a useful construct in the study of social withdrawal within other psychiatric conditions. Considering the complexity of the hikikomori group itself, further work should explore this relationship in detail, utilizing standardized diagnostic measures to investigate their mental status.

## Data Availability Statement

The raw data supporting the conclusions of this article will be made available by the authors, without undue reservation.

## Ethics Statement

The studies involving human participants were reviewed and approved by the Research Ethical Committee at Shanghai Mental Health Center. Written informed consent to participate in this study was provided by the participants.

## Author Contributions

YS contributed to the conception of the study and reviewed and approved the final version of the manuscript. DF performed the investigation. XH contributed to analysis and manuscript preparation. All authors contributed to the article and approved the submitted version.

## Funding

This work was funded by Quantitative Research of Intervention Strategies for Schizophrenia Recurrence (grant number: 19411950800), Health Law Research Association Program of Shanghai Law Society (grant number: 2020WF03), Shanghai Clinical Research Center for Mental Health (grant number: 19MC1911100), and Excellent Talent Training Program of Three Year Action Plan on Public Health of Shanghai (2020-2022) (grant number: GWV-10.2-XD27).

## Conflict of Interest

The authors declare that the research was conducted in the absence of any commercial or financial relationships that could be construed as a potential conflict of interest.

## Publisher's Note

All claims expressed in this article are solely those of the authors and do not necessarily represent those of their affiliated organizations, or those of the publisher, the editors and the reviewers. Any product that may be evaluated in this article, or claim that may be made by its manufacturer, is not guaranteed or endorsed by the publisher.
